# Th17 can regulate silica-induced lung inflammation through an IL-1β-dependent mechanism

**DOI:** 10.1111/jcmm.12341

**Published:** 2014-08-05

**Authors:** Laiyu Song, Dong Weng, Wujing Dai, Wen Tang, Shi Chen, Chao Li, Ying Chen, Fangwei Liu, Jie Chen

**Affiliations:** a Division of Pneumoconiosis, School of Public Health, China Medical UniversityShenyang, China; b Department of Clinical Immunology, Dalian Medical UniversityDalian, China; c Department of Respiratory Medicine, Shanghai Pulmonary Hospital, Tongji University School of MedicineShanghai, China

**Keywords:** silicosis, lung Inflammation, IL-1β, Th17

## Abstract

Silicosis is an occupational lung disease caused by the inhalation of silica dust and characterized by lung inflammation and fibrosis. Interleukin (IL)-1β is induced by silica and functions as the key pro-inflammatory cytokine in this process. The Th17 response, which is induced by IL-1β, has been reported very important in chronic human lung inflammatory diseases. To elucidate the underlying mechanisms of IL-1β and IL-17 in silicosis, we used anakinra and an anti-IL-17 monoclonal antibody (mAb) to block the receptor of IL-1β (IL-RI) and IL-17, respectively, in a mouse model of silicosis. We observed increased IL-1β expression and an enhanced Th17 response after silica instillation. Treatment with an IL-1 type I receptor (IL-1RI) antagonist anakinra substantially decreased silica-induced lung inflammation and the Th17 response. Lung inflammation and the accumulation of inflammatory cells were attenuated in the IL-17-neutralized silicosis group. IL-17 may promote lung inflammation by modulating the differentiation of Th1 and regulatory T cells (Tregs) and by regulating the production of IL-22 and IL-1β during the lung inflammation of silicosis. Silica may induce IL-1β production from alveolar macrophages and promote inflammation by initiating a Th17 response *via* an IL-1β/IL-1RI-dependent mechanism. The Th17 response could induce lung inflammation during the pathogenesis of silicosis by regulating the homoeostasis of the Th immune responses and affecting the production of IL-22 and IL-1β. This study describes a potentially important inflammatory mechanism of silicosis that may bring about novel therapies for this inflammatory and fibrotic disease.

## Introduction

Silica dioxide is the major component of most types of rocks, sand and soil, and silica dust exists abundantly in many industries related to those materials [1]. Inhalation of silica dust in working environment induces silicosis, an occupational lung disease characterized by lung inflammation and fibrosis [2]. Alveolar macrophages, the first defence against foreign substances, uptake inhaled silica and become activated to release several pro-inflammatory cytokines and chemokines that initiate a series of inflammatory reactions [3]. This silica-induced inflammation combines with repair processes, including proliferation of fibroblasts, deposition of collagen and consequent lung fibrosis. The progression of fibrosis is dependent on the development of inflammation [4]. By investigating the underlying mechanism of this inflammation, the mechanism of silicosis can be clarified, and novel approaches may result for preventing and curing this inflammatory and fibrotic disease.

Silica inhalation is associated with the activation of alveolar macrophages, a burst of inflammatory mediators, the influx of inflammatory cells, and the initiation of adaptive immune responses. Silica activates the macrophage NALP3 inflammasome to produce pro-interleukin (IL)-1β [5]. Caspase-1 in macrophages converts pro-IL-1β to its active and secreted form [6]. In addition, activated alveolar macrophages can initiate an effective T cell immune response. The pro-inflammatory cytokine, IL-1β, is a strong promoter of Th17 cell differentiation [7]. Th17 cells and IL-17 (also termed IL-17A) usually function during the inflammatory stage and induce inflammation by recruiting neutrophils and other cytokines [8]. We demonstrated recently that the Th17 response increases significantly during the early lung inflammation of silicosis, suggesting that the Th17 response may play a crucial role in the inflammation of silicosis [9]. Although the effect of silica on alveolar macrophages has been demonstrated, few studies have investigated the inflammation process occurring after alveolar macrophages are activated.

In this study, we used a mouse model to evaluate the initiation of silicosis. To investigate the underlying mechanism of silica-induced lung inflammation, we exposed mice to anakinra, an IL-1 receptor antagonist (IL-1Ra), and assessed the effect of IL-1β in the subsequent inflammatory response. We observed Th17 cell differentiation initiated by IL-1β *via* the IL-1 type I receptor (IL-1RI) at the beginning of the adaptive immune response. This study demonstrates the important role of IL-17 in lung inflammation and suggests a potential underlying mechanism.

## Materials and methods

### Animals

Female C57BL/6 mice were purchased from the Shanghai Laboratory Animal Center (Shanghai, China) at 6–8 weeks of age. All animals were housed in a specific pathogen-free environment and were maintained on standard mouse chow at an environmental temperature of 24 ± 1°C and a 12/12 h light/dark cycle with water *ad libitum*. All animal experiments were approved by the Animal Care and Use Committee of China Medical University (permit number, CMU62043013), which complies with the National Institutes of Health Guide for the Care and Use of Laboratory Animals.

### Reagents

Natural crystalline silica particles (Min-U-Sil 5 ground silica; size distribution: 97% <5 μm diameter, 80% <3 μm diameter; median diameter 1.4 μm) were obtained from the U.S. Silica Company (Frederick, MD, USA) [10]. Silica particles were boiled in 1 N HCl to remove endotoxins. Before use, the suspension was autoclaved and then sonicated as described previously [11]. To block IL-17, mice were treated with antimouse IL-17 neutralizing mAb (clone eBioMM17F3; eBioscience, San Diego, CA, USA) 100 μg intraperitoneally (i.p.)once a week since 1 day before silica exposure until killed. Control mice were administered rat IgG1 (BioLegend, San Diego, CA, USA) once a week since 1 day before silica exposure until killed [12]. To block the IL-1β receptor IL-1RI, mice were administered anakinra (1 mg; Biovitrum, Stockholm, Sweden) in sterile saline (i.p. 100 μl in total) twice daily since 1 day before silica exposure until killed [13]. Control mice were administered sterile saline alone (i.p. 100 μl in total) twice daily since 1 day before silica exposure until killed.

### Mouse model of silicosis

All 126 C57BL/6 mice were randomly divided into six groups (*n* = 21), as follows: (1) exposure to silica by direct oral-tracheal instillation of 50 μl aqueous suspensions of 2.5 mg silica crystals in sterile saline (silica group); (2) direct oral-tracheal instillation of 50 μl sterile saline (saline group); (3) direct oral-tracheal instillation of 2.5 mg silica dust 1 day after the first i.p. administration of 1 mg anakinra in 50 μl sterile saline (anakinra + silica group); (4) direct oral-tracheal instillation of 2.5 mg silica dust 1 day after the first i.p. administration of 50 μl sterile saline (saline + silica group); (5) direct oral-tracheal instillation of 2.5 mg silica dust 1 day after the first i.p. administration of 100 μg of anti-IL-17 mAb (anti-IL-17 mAb + silica group) (6) direct oral-tracheal instillation of 2.5 mg silica dust 1 day after the first i.p. administration of 100 μg control Ab (IgG1; control Ab + silica group). Mice exposed to silica underwent direct oral-tracheal instillation of aqueous suspensions of silica crystals (Min-U-Sil 5), as described previously [14]. Mice were killed on days 1, 4 and 11 after oral-tracheal instillation (Table S1). Bronchoalveolar lavage fluid (BALF) was obtained by cannulating the trachea and then injecting and retrieving 1 ml aliquots of PBS three times [15]. The BALF was centrifuged at 920 × ***g*** and 4°C for 8 min. After lysis of red blood cells (RBCs), the BALF cell pellet was washed and re-suspended in PBS. Total cell counts were determined using standard haematological procedures. After cytospin preparation, BALF was stained using the Wright-Giemsa method. Macrophages, neutrophils and lymphocytes were identified from fields of 200 cells using standard morphologic criteria [16].

### Isolation and culture of alveolar macrophages

Alveolar macrophages were isolated by incubating total BALF cells in 24-well plates at a density of 5 × 10^5^ cells/ml for 2 hrs and retaining adherent cells, as reported previously [17]. The adherent alveolar macrophages then were used as macrophages and incubated in RPMI 1640 supplemented with 10% foetal bovine serum (FBS), penicillin and streptomycin for 24 hrs [18]. Cell-free supernatants and macrophages were collected and frozen −80°C separately for subsequent analysis.

### Pathological examination

Lung tissue was fixed in 4% paraformaldehyde in PBS. Tissues were embedded in paraffin, cut into 6-μm-thick sections, and stained with haematoxylin and eosin. Lung morphology was visualized using an Olympus BX51 microscope at 200× magnification.

### Isolation of hilar lymph nodes and spleen cells

Hilar lymph nodes (HLNs) were harvested, dissected by needles quickly to little pieces, and digested with 0.25% trypsin for 5 min. at 37°C. The digestion reaction was terminated by the addition of 3% FBS in PBS, and samples were centrifuged at 920 × ***g*** and 4°C for 8 min. The HLNs cell pellet was washed and re-suspended in PBS. Spleens were removed, grinded and mechanically dissociated in cold PBS. After lysis of RBCs, spleen cells were washed and re-suspended in PBS.

### Cytokine analysis by ELISA

After bronchoalveolar fluid lavage, lung tissues were homogenized in nitrogen, and total proteins were extracted using a lysis buffer with phenylmethanesulfonyl fluoride. Samples were centrifuged at 4°C, and supernatants were stored at −80°C for subsequent analysis. Cytokines in the lung homogenate supernatants were analysed for IL-1β and IL-17 using ELISA, according to the manufacturer's instructions (bdbiosciences.com).

### RNA extraction and real-time reverse transcription (RT)-polymerase chain reaction (PCR)

Total RNA was extracted from lung and spleen homogenates using TRIzol reagent (Invitrogen, Carlsbad, CA, USA), according to the manufacturer's protocol. Total lung RNA (2 μg) and spleen RNA (1 μg) samples were reverse-transcribed (RT) separately in 20-μl volumes using a program of 37°C for 15 min. and 85°C for 5 sec. using ABI 2720 (Applied Biosystems, Foster City, CA, USA). Primers and TaqMan probes were designed using Primer3 (http://bioinfo.ut.ee/primer3-0.4.0/primer3/), and sequences were submitted to BLAST (http://blast.ncbi.nlm.nih.gov/Blast.cgi). Real-time RT-polymerase chain reaction (PCR) then was performed with a premix Ex Taq RT-PCR kit (DRR039A; Takara, Dalian, China) or a SYBR Premix Ex Taq II RT-PCR kit (DRR081A; Takara). A total of 2 μl of cDNA was used in each 25-μl PCR volume. Each sample was assayed in triplicate. Differences in amplification efficiencies between the target and housekeeping genes were identified by comparing standard curve slopes. Real-Time PCR was performed with ABI 7500 (Applied Biosystems) according to the following program: (*i*) TaqMan: 95°C for 30 sec., 40 cycles of 95°C for 5 sec., and 62°C for 34 sec., or (*ii*) SYBR Green: 95°C for 30 sec., 40 cycles of 95°C for 5 sec., and 60°C for 34 sec. PCR analyses were performed with ABI 7500 system software.

### Flow cytometry analysis

The cells from spleen and HLNs were stimulated with phorbol-12-myristate-13-acetate (50 ng/ml) and ionomycin (1 mg/ml; Sigma-Aldrich, Palo Alto, CA, USA) for 4 hrs, and monensin (BD GolgiStop Protein Transport Inhibitor) was added, as described previously [14]. Fc receptors (FcRs) were blocked with purified rat antimouse CD16/CD32 (BD Pharmingen, San Jose, CA, USA). Cells were fixed and permeabilized using standard reagents, according to the manufacturer's protocols (BD Biosciences, Franklin Lakes, NJ, USA). Cells then were stained with an antimouse CD4 PerCP-Cy5.5 clone: RM4-5; an antimouse IL-17 PE clone: TC11-18H10.1; an antimouse Foxp3 Alexa Fluor 647 clone: MF23; and an antimouse interferon gamma (IFN-γ) Alexa Fluor 488 Clone: XMG1.2 (BD Pharmingen). Cells then were analysed using a FACSCanto II flow cytometer (BD Biosciences). Dead cells and silica particles were gated out depending on forward scattering and side scattering. Cells were analysed using Diva software (http://modb.oce.ulg.ac.be/mediawiki/index.php/DIVA).

### Statistics

SPSS 16.0 software was used to conduct statistical analyses. All values were represented as means ± SEM. The differences between values were evaluated by one-way anova followed by pair-wise comparisons with the Student-Newman–Keuls test. Correlations between Th17 and IL-1β in alveolar macrophages were assessed using Pearson's correlation coefficients. *P* < 0.05 was considered statistically significant.

## Results

### Silica may activate alveolar macrophages to secrete IL-1β and increase Th17 responses in the inflammatory stage of silicosis

**Fig. 1:**
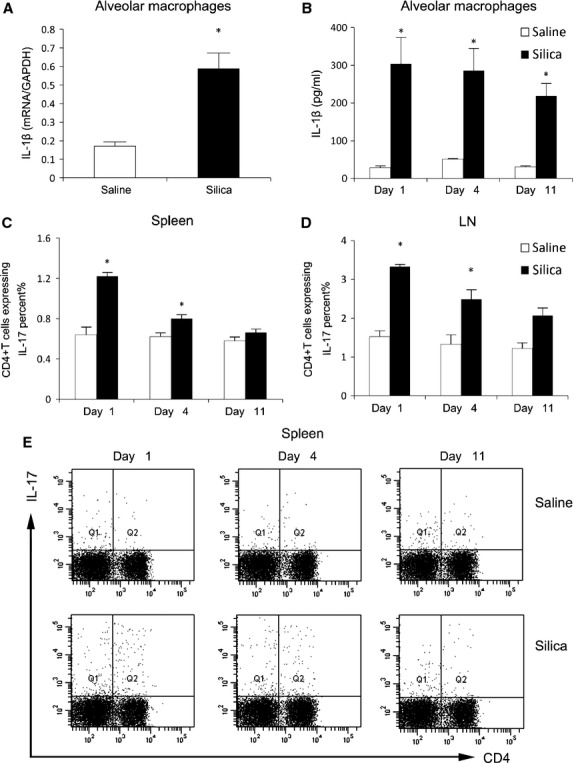
Silica may activate alveolar macrophages to secrete IL-1β and increase the Th17 response in the inflammatory stage of silicosis. IL-1β mRNA expression in alveolar macrophages (**A**) was assayed by real-time RT-PCR using the −ΔΔCt method (*n* = 3). Levels of IL-1β (**B**) in the supernatants of cultured alveolar macrophages were detected by ELISA (*n* = 4). Th17 cells in the spleen (**C** and **E**) and hilar lymph nodes (HLNs; **D**) were calculated by flow cytometry, and the percentage of CD4 + IL-17 + T cells was plotted (**C** and **D**; *n* = 4; *P < 0.05 *versus* the saline control group).

To better understand the initiation of silicosis, we examined alveolar macrophages in a C57BL/6 mouse model. IL-1β mRNA expression in alveolar macrophages was increased significantly after 1 day of silica exposure (*P* < 0.05; Fig. 1A). In contrast, IL-17 mRNA expression in alveolar macrophages was not affected significantly after silica treatment (data not shown). We then examined IL-1β protein expression from the supernatants of cultured alveolar macrophages isolated from our mouse model of silicosis. Alveolar macrophages from silica-treated mice secreted significantly more IL-1β than saline-treated mice at three early time-points (*P* < 0.05; Fig. 1B). The increased IL-1β expression and secretion induced by silica in this experiment confirmed the activation of alveolar macrophages *in vivo* demonstrated by others [19].

We then examined the Th17 (CD4+IL-17+) cell response, which mediates early-stage inflammation. Th17 cell increased significantly in the spleen and HLNs of silica-treated mice compared with saline-treated controls on days 1 and 4 after silica treated, with the most substantial increase on day 1 (*P* < 0.05; Fig. 1C–E). Fluorescence-activated cell sorting (FACS) indicated that the differentiated Th17 cells peaked immediately after silica exposure and decreased gradually thereafter (*P* < 0.05; Fig. 1C–E). These data suggest that Th17 cells might mark the beginning of the adaptive immune response in silica-induced lung inflammation.

Th1 cells usually function as inflammatory cells by secreting IFN-γ and IL-12. The Th1 cells in the spleen and HLNs were increased in silica-treated mice compared with saline-treated controls (*P* < 0.05; Fig. S1A and B). We observed a linear relationship between IL-1β protein expression in alveolar macrophages and the percentage of Th17 cells in the spleen (*P* < 0.001; Fig. S1C). In contrast, no relationship between Th1 cells and IL-1β in alveolar macrophages was identified (Fig. S1D). These results suggest a connection between Th17 cells and IL-1β secreted by activated alveolar macrophages *in vivo*.

### Silica-induced IL-1β may enhance lung inflammation by initiating Th17 responses through the IL-1 type I receptor

**Fig. 2:**
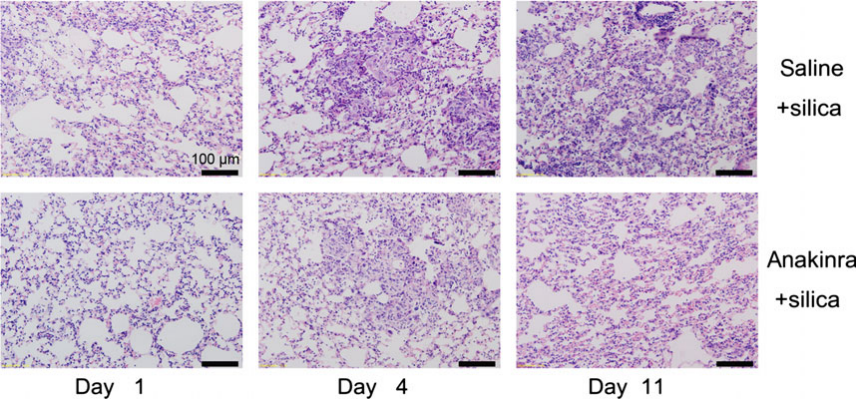
Blockade of the IL-1β receptor by anakinra decreases silica-induced lung inflammation. Histopathological changes in the lung tissue of mice treated by silica instillation (×200). The scale on the graph above is 100 μm; data were gathered on days 1, 4 and 11 after silica exposure (day 0). Lung sections were stained with haematoxylin and eosin. The degree of inflammation was assessed by histological analyses of six random fields per sample (*n* = 5 per group).

Pathological examination indicated fewer infiltrated cells in the anakinra + silica group than in the saline+silica group. Alveolar wall change in the anakinra + silica group also was less than that of the saline + silica group. Anakinra significantly decreased silica-induced lung inflammation (Fig. 2). Total cells and neutrophils in BALF decreased significantly in the anakinra + silica group on days 1 and 4 (*P* < 0.05; Fig. S2A and B). Anakinra decreased the accumulation of lymphocytes on day 4 and macrophages on days 1 and 11 (*P* < 0.05; Fig. S2C and D). Our analysis of inflammatory cells in the BALF confirmed the status of attenuated inflammation in the anakinra + silica group. A decreased neutrophils in the anakinra + silica group suggested that neutrophilic inflammation might depend on the Th17 response in the early stage. Our previous study and the results presented here demonstrate that IL-1β can modulate the Th17 response in the inflammatory stage of silicosis [9]. We suggest that IL-1β enhances early lung inflammation by inducing Th17 cell differentiation.

**Fig. 3:**
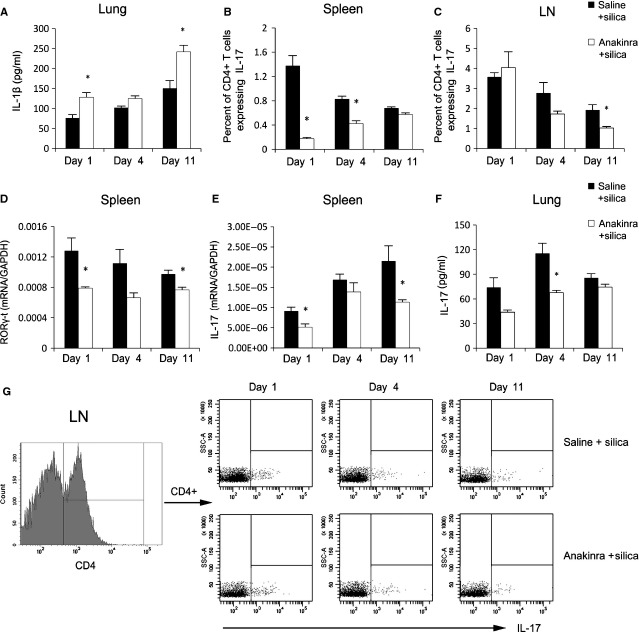
Silica-induced IL-1β may enhance lung inflammation by initiating Th17 response through IL-1RI. The IL-1β protein level in lung tissue (**A**) was examined by ELISA (*n* = 4). Th17 cells in the spleen (**B**) and hilar lymph nodes (HLNs; **C** and **G**) were calculated by flow cytometry, and the percentage of CD4 + IL-17 + T cells was plotted (**B** and **C**; *n* = 4). RORγt (**D**) and IL-17 mRNA levels (**E**) in the spleen were assayed by real-time RT-PCR using the −ΔΔCt method (*n* = 4). IL-17 protein expression in lung tissue (**F**) was examined by ELISA (*n* = 4; *P < 0.05 *versus* the saline + silica control group).

To investigate the underlying mechanism of IL-1β regulating Th17 cell differentiation in silicosis, we disabled silica-induced IL-1β using anakinra (1 mg/mouse). Anakinra blocks the biological activity of IL-1, particularly IL-1β, by competitively inhibiting IL-1 binding to the IL-1RI, which is expressed in a wide variety of tissues and organs [20]. We examined IL-1β protein expression in homogenized lung tissue on days 1, 4 and 11 after anakinra and silica treatments. IL-1β protein expression in the anakinra + silica group increased significantly on days 1 and 11 compared with the saline + silica group (*P* < 0.05; Fig. 3A), which confirmed that anakinra inhibits IL-1β binding to its receptor [21]. We then examined the Th17 response in the presence of anakinra. In our mouse model of silicosis, anakinra significantly decreased the percentage of Th17 cells in the spleen on days 1 and 4 (*P* < 0.05; Fig. 3B). Anakinra also decreased the percentage of Th17 cells in lymphocytes on days 4 and 11 (Fig. 3C) The mRNA expression levels of IL-17 and the transcription factor retinoic acid-receptor-related orphan nuclear receptor γ-t (RORγ-t) in the spleen decreased in the anakinra + silica group on days 1, 4 and 11 (*P* < 0.05; Fig. 3D and 3E). The protein level of IL-17 in lung tissues decreased in the anakinra + silica group compared with that in the saline + silica group, particularly on days 1 and 4 (Fig. 3F). FACS also indicated the decreased percentage of Th17 cells in lymphocytes (Fig. 3G). These data suggest that IL-1β could regulate the differentiation of Th17 cells and the expression of IL-17 through IL-1RI. Anakinra did not significantly influence the Th1 response (Fig. S3).

### Th17 responses play a crucial role in promoting silica-induced lung inflammation

**Fig. 4:**
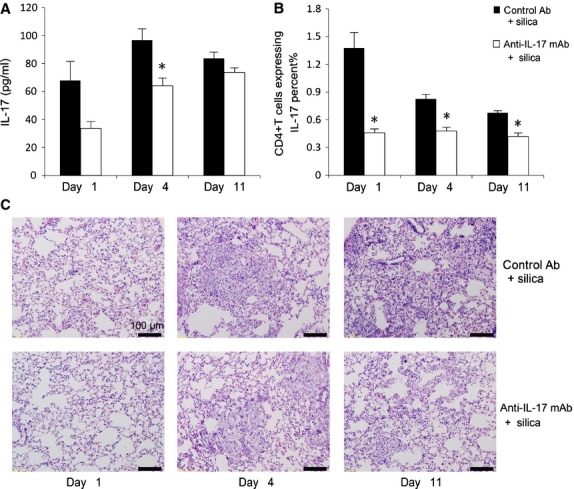
Th17 responses play a crucial role in promoting silica-induced lung inflammation. IL-17 protein expression in lung tissue (**A**) was examined by ELISA (*n* = 4). Th17 cells in the spleen (**B**) were calculated by flow cytometry, and the percentage of CD4 + IL-17 + T cells was plotted (*n* = 4). Neutralization of IL-17 decreased silica-induced early lung inflammation. Histopathological changes in lung tissues of mice treated by silica instillation (haematoxylin and eosin staining; ×200; **C**). The scale on the graph above is 100 μm. Data were collected on days 1, 4 and 11 after silica exposure. The degree of inflammation was assessed by the histological analysis of six random fields per sample (*n* = 5 per group; *P < 0.05 *versus* the control Ab + silica group).

**Fig. 5:**
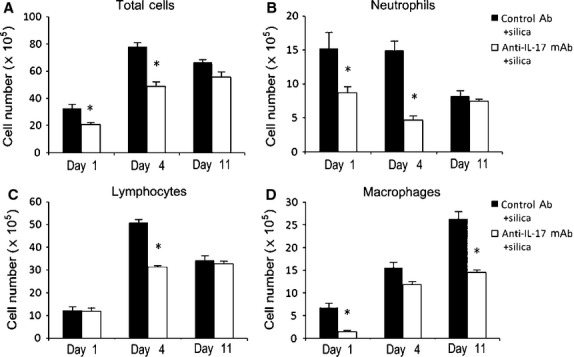
The Th17 response promotes the accumulation of inflammatory cells in BALF. Total cells (**A**), neutrophils (**B**), lymphocytes (**C**) and macrophages (**D**) in BALF were counted using Giemsa staining (*n* = 4). Results are represented as means ± SEM (*P < 0.05 *versus* the control Ab + silica group).

To assess the function of the IL-17/Th17 response in the inflammatory stage of silicosis, we examined the lung response to IL-17 neutralization. An anti-IL-17 mAb effectively neutralized IL-17 in the lung on days 1 and 4, significantly at day 4 (*P* < 0.05; Fig. 4A). Th17 cells in the spleen were decreased significantly in the anti-IL-17 mAb+silica group compared with Th17 cells in the control Ab+silica group (*P* < 0.05; Fig. 4B). These results demonstrated neutralization of IL-17 in mice treated with an anti-IL-17 mAb. Silica-induced lung inflammation was limited in the group administered anti-IL-17 mAb compared with mice administered control Ab (Fig. 4C). We then examined the accumulation of inflammatory cells (*i.e*., total cells, neutrophils, lymphocytes and macrophages) in BALF. Neutralization of IL-17 downregulated the total cell numbers in BALF significantly on days 1 and 4 (*P* < 0.05; Fig. 5A). Neutrophils in the anti-IL-17 mAb + silica group decreased significantly compared with those in the saline + silica group on days 1 and 4 (*P* < 0.05; Fig. 5B). Lymphocytes and macrophages in the anti-IL-17 mAb+silica group both decreased to varying degrees (Fig. 5C and D). These results support key functions for IL-17/Th17 in the development of lung inflammation in silicosis.

### IL-17 may regulate silica-induced lung inflammation by modulating the Th immune balance

**Fig. 6:**
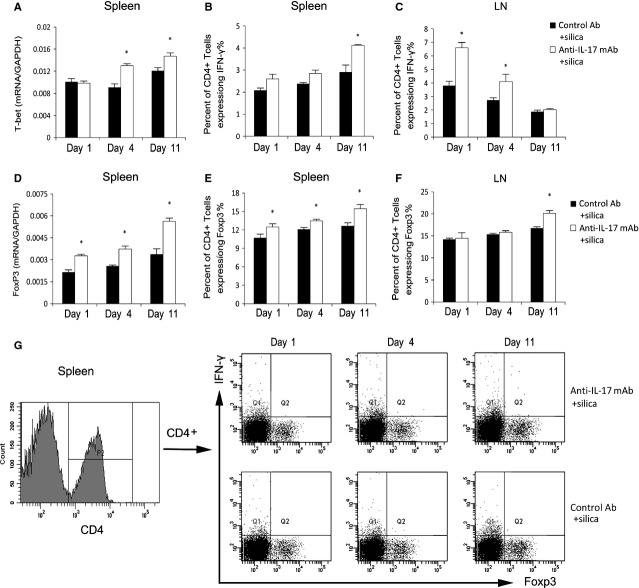
IL-17 may regulate silica-induced lung inflammation by modulating the Th immune balance. The levels of T-bet (**A**) and Foxp3 mRNA (**D**) in the spleen were assayed by real-time RT-PCR using the −ΔΔCt method (*n* = 4). Th1 cells in the spleen (**B** and **G**) and hilar lymph nodes (HLNs; **C**) were calculated by flow cytometry, and the percentage of CD4 + IFN-γ+ T cells was plotted (**B** and **C**; *n* = 4). Tregs in the spleen (**E** and **G**) and HLNs (**F**) were calculated by flow cytometry, and the percentage of CD4 + IFN-γ+ T cells was plotted (**E** and **F**; *n* = 4). Results are represented as means ± SEM (*P < 0.05 *versus* the control Ab + silica group)).

The Th immune response is very important in the progress of lung inflammation [22]. The massive expansion of the Th17 cell population can create an imbalance between effector T cells (such as Th1 cells) and regulatory T cells (Tregs), which determine the outcome of adaptive immune responses [23]. We assessed the effects of IL-17 on the T cell immune response to test whether IL-17 could promote the lung inflammation of silicosis by influencing related immune responses. We examined T-bet, the key transforming growth factor of Th1 cells. IL-17 neutralization significantly increased T-bet mRNA expression in our silicosis model on days 4 and 11 (*P* < 0.05; Fig. 6A). CD4+IFN-γ+ Th1 cells in the spleen were increased significantly in the anti-IL-17 mAb+silica group compared with those in the control Ab + silica group on day 11 (*P* < 0.05; Fig. 6B). In the HLNs of anti-IL-17 mAb + silica-treated mice, CD4+IFN-γ+ Th1 cells were significantly increased in the anti-IL-17 mAb + silica group compared with those in the control Ab + silica group on days 1 and 4 (*P* < 0.05; Fig. 6C). These data suggest that the Th1 immune response is increased in the silicosis mouse model treated with anti-IL-17 mAb.

A previous report described the regulatory roles of Tregs and Th17 cells in the process of obstructive pulmonary disease [24]. Compared with mice administered control Ab, IL-17-neutralized silicosis mice displayed significantly increased Foxp3 mRNA expression at all three time-points (*P* < 0.05; Fig. 6D). FACS indicated that spleen and lymphocyte Tregs (CD4+Foxp3+) also were increased in the anti-IL-17 mAb + silica group compared with those in the control Ab+silica group (*P* < 0.05; Fig. 6E–G). These data demonstrate the increased Treg response in IL-17-neutralized silicosis mice.

### Th17 may promote silica-induced lung inflammation by regulating the production of IL-22 and IL-1β

**Fig. 7:**
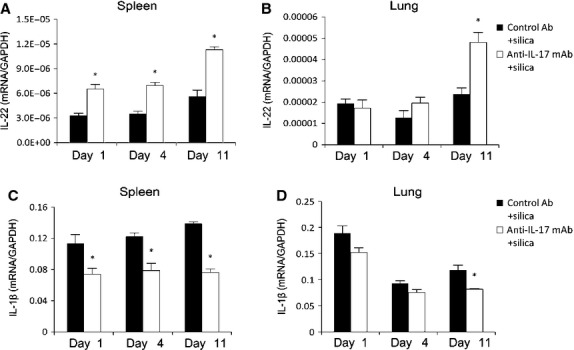
Th17 may promote silica-induced lung inflammation and silicosis by regulating the production of IL-22 and IL-1β. The expression levels of IL-22 mRNA in spleen (**A**) and lung (**B**) tissues were assayed by real-time RT-PCR using the −ΔΔCt method (*n* = 4). The expression levels of IL-1β mRNA in spleen (**C**) and lung (**D**) tissues were assayed by real-time RT-PCR using the −ΔΔCt method (*n* = 4; *P < 0.05 *versus* the control Ab + silica group)).

Several studies previously demonstrated the pro-inflammatory and/or pathological properties of IL-22 [25]. Others have reported that IL-22 is an anti-inflammatory cytokine with suppressive effects in asthma [26]. IL-22 mRNA expression in the spleens of anti-IL-17 mAb+silica-treated mice increased significantly compared with IL-22 expression in the control Ab+silica group at three time-points (Fig. 7A). IL-22 mRNA expression in the lungs of anti-IL-17 mAb+silica-treated mice also increased relative to controls (Fig. 7B). IL-17 neutralization may increase IL-22 expression in the lung inflammation of silicosis.

Although many previous reports, including those from our laboratory, have demonstrated the effects of IL-1β on Th17 and IL-17 [8], the influence of IL-17 on IL-1β expression remained unclear. Thus, we examined the effects of IL-17 on IL-1β expression in this model. The mRNA expression of IL-1β in spleens decreased significantly after IL-17 neutralization (Fig. 7C) in our mouse model. The IL-1β mRNA expression in lung tissue also decreased significantly in the anti-IL-17 mAb+silica group (Fig. 7D). These data suggest that IL-17 can influence IL-1β expression in the lung inflammation of silicosis.

## Discussion

Silicosis is an occupational lung disease caused by the inhalation of silica dust and characterized by lung inflammation and fibrosis. In the radiological findings, silica will induce small particulated shadows at the upper and middle lobes of the lungs, which is different from the liner shadows at middle to lower lungs caused by asbestosis. The lung inflammations caused by silica or asbestos are quite different as well. Silica often induces localized nodular pulmonary inflammation combined with interstitial inflammation sometimes. But the inflammation induced by asbestosis is a diffused interstitial pulmonary inflammation. The silica-induced lung inflammation is typically initiated by an interaction between silica particles and antigen presenting cells. Macrophages, the key components of the innate immune response, have been recognized as the first defence against foreign substances [27]. Silica can induce the abundant secretion of IL-1β from macrophages [28]. Innate immune processes initiate adaptive immune responses and lead ultimately to lung fibrosis. However, the mechanism of silica-induced early lung inflammation that follows the activation of alveolar macrophages has not been examined sufficiently to date. In this study, we used C57BL/6 mice to evaluate the underlying mechanism of lung inflammation in silicosis. Anakinra and an anti-IL-17 mAb were administered to neutralize the function of IL-1β and the Th17 response. Anakinra significantly decreased silica-induced lung inflammation. The pro-inflammatory function of IL-1β may depend, in part, on the increased Th17 response.

We first examined the initiation of silica-induced inflammation. After silica treatment, increased IL-1β mRNA and protein expression levels were measured in alveolar macrophages, confirming an early reaction between silica and alveolar macrophages. FACS indicated that silica exposure increased the percentage of Th17 cells in the spleen and HLNs, especially at the earliest time-point after silica exposure. Instead of its pro-inflammatory function, IL-1β generally functioned as a strong promoter of Th17 differentiation during the early stage of inflammation [29]. Previous studies have suggested that IL-17 secretion is promoted by IL-1β in autoimmune and allergic lung diseases [30,31]. We also demonstrated previously that IL-1β is a key promoter of Th17 cell differentiation [19]. Therefore, we speculated that IL-1β might bridge the activation of alveolar macrophages and subsequent inflammation by inducing a Th17 response in silica-induced lung inflammation. We identified a correlation between Th17 cells and IL-1β protein. FACS also suggested an augmented Th1 response in silicosis, but the connection between the Th1 response and IL-1β protein level was not obvious.

Human Th17 polarization induced by Mycobacterium tuberculosis was IL-1β dependent [32]. The mode by which IL-1β manipulates Th17 cell differentiation in silicosis remains unclear. A previous report suggested that IL-1R must be functional for a Th17 response in humans [33]. Therefore, we used anakinra, a recombinant nonglycosylated human IL-1Ra that can bind competitively to the IL-1RI to block the biological activity of silica-induced IL-1β. Cigarette smoke-induced accumulation of inflammation and CD4+ T cells was significantly impaired in IL-1RI knock-out (KO) mice [34]. Our observation of decreased inflammation in the anakinra + silica group suggested that anakinra was a useful drug to limit the lung inflammation induced by silica. FACS and real-time RT-PCR confirmed that anakinra decreased the Th17 response and Th17 cell differentiation in silica-induced lung inflammation. These results demonstrated that IL-1β could modulate Th17 cell differentiation *via* IL-1RI in our model. The modest difference in Th1 responses between the anakinra + silica group and the saline + silica group demonstrated the minor contribution of IL-1β in the Th1 response during the lung inflammation of silicosis. The decreased Th17 immune response also might contribute to the finding of fewer inflammatory cells in the anakinra + silica group.

In a number of inflammatory disease models, Th17 and/or IL-17 mediate inflammation by recruiting neutrophils and regulating other inflammatory cells [35]. Yoshida found IL-17A gene-KO mice failed to develop mature granulomas in the Mycobacterium bovis bacille Calmette-Guerin-infected lung [36]. Th17 responses have been detected previously in lung inflammation and fibrosis [37]. To test the pro-inflammatory function of IL-17 and investigate its underlying mechanism in silica-induced lung inflammation, we neutralized IL-17 by administrating an anti-IL-17 mAb. The decreased protein level of IL-17 and decreased Th17 cells in the lung and spleen verified the successful neutralization of IL-17 by the anti-IL-17 mAb. The inflammatory cells in BALF collected from our mouse model confirmed previous reports that the Th17 response was a critical recruiter of neutrophils in lung inflammation of silicosis [35]. IL-17 also could influence the accumulation of macrophages and lymphocytes in silica-induced lung inflammation. IL-17R-deficient animals (IL-17R(-/-)) developed reduced neutrophil influx and injury during the early lung response to silica [14]. Our BALF analyses and pathological examinations demonstrated the pro-inflammatory function of IL-17 during lung inflammation in our experimental mouse model of silicosis.

The regulatory function of IL-17 in the Th1 response was unclear; this response is dominated by macrophage activation and granuloma formation during lung inflammation in a mouse model of silicosis [4,38]. Increased Th1 lymphocytes and IFN-γ have been identified in lymph nodes and lung tissue during the lung inflammation of silicosis [39]. An interaction between Th17 and Th1 responses also has been reported [40,41]. We observed that in IL-17-neutralized mice, the Th1 response increased in silica-induced lung inflammation, confirming the regulatory function of IL-17 in the Th1 response. Instead of regulating the Th1 response, Th17 responses could influence the development of Tregs. Tregs modulate Th immune responses and are critical to reducing allergic lung inflammation [42]. The increased Tregs in the IL-17-neutralized silicosis group confirmed the inhibitory function of Th17 responses on the generation of Tregs [43]. IL-17 may modulate the balance of Th immune responses to influence silica-induced lung inflammation.

Interleukin-22, which is recognized as a member of the IL-10 cytokine family, can be produced by Th22, Th1, and Th17 cells; classical and non-classical (NK-22) NK cells; NKT cells; and lymphoid tissue inducer cells [44]. The coexpression and cooperative actions of IL-17 and IL-22 have been reported previously [45,46]. The potential discrepancy between different experiments may be due to different mice model. In a similar mice model of lung inflammation and fibrosis induced by bleomycin, IL-22 expression would be manipulated by IL-17A and protect the lung tissue in the absence of IL-17A [47]. In the present study, we demonstrated an increased IL-22 level in IL-17-neutralized silicosis mice. The protective function of IL-22 in lung inflammation has been suggested previously [26]. Thus, we proposed that decreased lung inflammation in the IL-17-neutralized group might contribute to an increased level of IL-22. We speculated that increased IL-22 might contribute to a compensatory increase in Th22 cells, NK-22 cells and other cells in the IL-17-neutralized mice model of silicosis. In a future study, we plan to investigate the cross-talk between IL-22 and IL-17 in our silicosis model.

Previous research has demonstrated that IL-1β mediates lung inflammation and that fibrosis is IL-17 dependent [48]. However, we observed decreased IL-1β in our IL-17-neutralized group, suggesting the importance of IL-17 in IL-1β expression. As the decreased lung inflammation and Th17 response in the IL-1Ra-treated group, we detect a positive interaction between the Th17 response and IL-1β expression in silica-induced lung inflammation. IL-1β secreted by alveolar macrophages can initiate an innate immune response by inducing a Th17 response in lung inflammation. The Th17 response subsequently can regulate lung inflammation by mediating feedback expression of IL-1β. The IL-22 and IL-1β mRNA expression in lung was not all as significantly as that in spleen. This interesting discrepancy between lung and spleen suggested the anti-IL-17mAb worked on spleen in front of working on lung. In this study, we choose the ways of cytokine neutralization and receptor blockage to study and intend to find a useful drug to relieve the inflammation of silicosis. Next, we will use the IL-17 and IL-1RI knock-out mouse to investigate the underlying molecular mechanism in this model.

In summary, our study demonstrated that alveolar macrophages could be activated by silica to secrete the pro-inflammatory cytokine, IL-1β. The production of IL-1β might initiate a Th17-dominant immune response through an IL-1RI-dependent mechanism. The Th17 response is a key to promoting silica-induced lung inflammation, potentially by regulating the homoeostasis of Th immune responses and the production of IL-22 and IL-1β. We also identified positive feedback between IL-1β and the Th17 response in our mouse model of silicosis. The present study describes a potentially important mechanism of silica-induced lung inflammation that may lead to novel therapies for silicosis.

## Supporting information

**Figure S1** Silica exposure may facilitate a Th1 response in C57/BL6 mice.

**Figure S2** Blockade of the IL-1β receptor by Anakinra decreases the accumulation of BALF inflammatory cells.

**Figure S3** IL-1β does not influence the Th1 immune response in the inflammatory stage of silicosis.

**Table S1** Animal grouping moulding and processing.

## References

[1] Wilson MS, Wynn TA (2009). Pulmonary fibrosis: pathogenesis, etiology and regulation. Mucosal Immunol.

[2] Kühlmann UC, Chwieralski CE, van den Brule S (2009). Modulation of cytokine production and silica-induced lung fibrosis by inhibitors of aminopeptidase N and of dipeptidyl peptidase-IV-related proteases. Life Sci.

[3] Joshi GN, Knecht DA (2013). Silica phagocytosis causes apoptosis and necrosis by different temporal and molecular pathways in alveolar macrophages. Apoptosis.

[4] Migliaccio CT, Buford MC, Jessop F (2008). The IL-4Ralpha pathway in macrophages and its potential role in silica-induced pulmonary fibrosis. J Leukoc Biol.

[5] Hornung V, Bauernfeind F, Halle A (2008). Silica crystals and aluminum salts activate the NALP3 inflammasome through phagosomal destabilization. Nat Immunol.

[6] Hu Y, Mao K, Zeng Y (2010). Tripartite-motif protein 30 negatively regulates NLRP3 inflammasome activation by modulating reactive oxygen species production. J Immunol.

[7] Chung Y, Chang SH, Martinez GJ (2009). Critical regulation of early Th17 cell differentiation by interleukin-1 signaling. Immunity.

[8] Pridgeon C, Bugeon L, Donnelly L (2011). Regulation of IL-17 in chronic inflammation in the human lung. Clin Sci.

[9] Song L, Weng D, Liu F (2012). Tregs promote the differentiation of Th17 cells in silica-induced lung fibrosis in mice. PLoS ONE.

[10] Herseth JI, Volden V, Schwarze PE (2008). IL-1beta differently involved in IL-8 and FGF-2 release in crystalline silica-treated lung cell co-cultures. Part Fibre Toxicol.

[11] Peeters PM, Perkins TN, Wouters EFM (2013). Silica induces NLRP3 inflammasome activation in human lung epithelial cells. Part Fibre Toxicology.

[12] Uyttenhove C, Van Snick J (2006). Development of an anti-IL-17A auto-vaccine that prevents experimental auto-immune encephalomyelitis. Eur J Immunol.

[13] Provoost S, Maes T, Pauwels NS (2011). NLRP3/caspase-1-independent IL-1beta production mediates diesel exhaust particle-induced pulmonary inflammation. J Immunol.

[14] Lo Re S, Dumoutier L, Couillin I (2010). IL-17A-producing gammadelta T and Th17 lymphocytes mediate lung inflammation but not fibrosis in experimental silicosis. J Immunol.

[15] Bang BR, Chun E, Shim EJ (2011). Alveolar macrophages modulate allergic inflammation in a murine model of asthma. Exp Mol Med.

[16] Yang T, Luo F, Shen Y (2012). Quercetin attenuates airway inflammation and mucus production induced by cigarette smoke in rats. Int Immunopharmacol.

[17] Kuroda E, Yamashita U (2003). Mechanisms of enhanced macrophage-mediated prostaglandin E2 production and its suppressive role in Th1 activation in Th2-dominant BALB/c mice. J Immunol.

[18] Mariani TJ, Arikan MC, Pierce RA (1999). Fibroblast tropoelastin and alpha-smooth-muscle actin expression are repressed by particulate-activated macrophage-derived tumor necrosis factor-alpha in experimental silicosis. Am J Respir Cell Mol Biol.

[19] Cassel SL, Eisenbarth SC, Iyer SS (2008). The Nalp3 inflammasome is essential for the development of silicosis. Proc Natl Acad Sci.

[20] Pazyar N, Feily A, Yaghoobi R (2012). An overview of interleukin-1 receptor antagonist, anakinra, in the treatment of cutaneous diseases. Curr Clin Pharmacol.

[21] Lindauer ML, Wong J, Iwakura Y (2009). Pulmonary inflammation triggered by ricin toxin requires macrophages and IL-1 signaling. J Immunol.

[22] Huaux F (2007). New developments in the understanding of immunology in silicosis. Curr Opin Allergy Clin Immunol.

[23] Shao S, He F, Yang Y (2012). Th17 cells in type 1 diabetes. Cell Immunol.

[24] Lane N, Robins RA, Corne J (2010). Regulation in chronic obstructive pulmonary disease: the role of regulatory T-cells and Th17 cells. Clin Sci.

[25] Zheng Y, Danilenko DM, Valdez P (2007). Interleukin-22, a T(H)17 cytokine, mediates IL-23-induced dermal inflammation and acanthosis. Nature.

[26] Pennino D, Bhavsar PK, Effner R (2013). IL-22 suppresses IFN-γ-mediated lung inflammation in asthmatic patients. J Allergy Clin Immunol.

[27] Franchi L, Muñoz-Planillo R, Núñez G (2012). Sensing and reacting to microbes through the inflammasomes. Nat Immunol.

[28] Hoffman HM, Wanderer AA (2010). Inflammasome and IL-1beta-mediated disorders. Curr Allergy Asthma Rep.

[29] Cascão R, Moura RA, Perpétuo I (2010). Identification of a cytokine network sustaining neutrophil and Th17 activation in untreated early rheumatoid arthritis. Arthritis Res Ther.

[30] Lalor SJ, Dungan LS, Sutton CE (2011). Caspase-1-processed cytokines IL-1beta and IL-18 promote IL-17 production by gammadelta and CD4 T cells that mediate autoimmunity. J Immunol.

[31] Zhu S, Qian Y (2012). IL-17/IL-17 receptor system in autoimmune disease: mechanisms and therapeutic potential. Clin Sci.

[32] Dinarello CA (2011). Interleukin-1 in the pathogenesis and treatment of inflammatory diseases. Blood.

[33] Lee WW, Kang SW, Choi J (2010). Regulating human Th17 cells *via* differential expression of IL-1 receptor. Blood.

[34] Pauwels NS, Bracke KR, Dupont LL (2011). Role of IL-1α and the Nlrp3/caspase-1/IL-1β axis in cigarette smoke-induced pulmonary inflammation and COPD. Eur Respir J.

[35] Nembrini C, Marsland BJ, Kopf M (2009). IL-17-producing T cells in lung immunity and inflammation. J Allergy Clin Immunol.

[36] Okamoto Yoshida Y, Umemura M, Yahagi A (2010). Essential role of IL-17A in the formation of a mycobacterial infection-induced granuloma in the lung. J Immunol.

[37] Simonian PL, Roark CL, Wehrmann F (2009). Th17-polarized immune response in a murine model of hypersensitivity pneumonitis and lung fibrosis. J Immunol.

[38] Davis GS, Pfeiffer LM, Hemenway DR (2000). Interferon-gamma production by specific lung lymphocyte phenotypes in silicosis in mice. Am J Respir Cell Mol Biol.

[39] Davis GS, Pfeiffer LM, Hemenway DR (1999). Expansion of interferon-gamma-producing lung lymphocytes in mouse silicosis. Am J Respir Cell Mol Biol.

[40] Dardalhon V, Korn T, Kuchroo VK (2008). Role of Th1 and Th17 cells in organ-specific autoimmunity. J Autoimmun.

[41] Rangachari M, Mauermann N, Marty RR (2006). T-bet negatively regulates autoimmune myocarditis by suppressing local production of interleukin 17. J Exp Med.

[42] Liu F, Liu J, Weng D (2010). CD4 + CD25 + Foxp3 + regulatory T cells depletion may attenuate the development of silica-induced lung fibrosis in mice. PLoS ONE.

[43] Bettelli E, Carrier Y, Gao W (2006). Reciprocal developmental pathways for the generation of pathogenic effector TH17 and regulatory T cells. Nature.

[44] Witte E, Witte K, Warszawska K (2010). Interleukin-22: a cytokine produced by T, NK and NKT cell subsets, with importance in the innate immune defense and tissue protection. Cytokine Growth Factor Rev.

[45] Besnard AG, Sabat R, Dumoutier L (2011). Dual Role of IL-22 in allergic airway inflammation and its cross-talk with IL-17A. Am J Respir Crit Care Med.

[46] Aujla SJ, Chan YR, Zheng M (2008). IL-22 mediates mucosal host defense against Gram-negative bacterial pneumonia. Nat Med.

[47] Sonnenberg GF, Nair MG, Kirn TJ (2010). Pathological *versus* protective functions of IL-22 in airway inflammation are regulated by IL-17A. J Exp Med.

[48] Wilson MS, Madala SK, Ramalingam TR (2010). Bleomycin and IL-1beta-mediated pulmonary fibrosis is IL-17A dependent. J Exp Med.

