# Supplementary figures and images for: Th17 can regulate silica-induced lung inflammation through an IL-1β-dependent mechanism
Source: J Cell Mol Med. 2014 Aug 5;18(9):1773–84. doi: 10.1111/jcmm.12341 (PMC4196653; doi:10.1111/jcmm.12341)

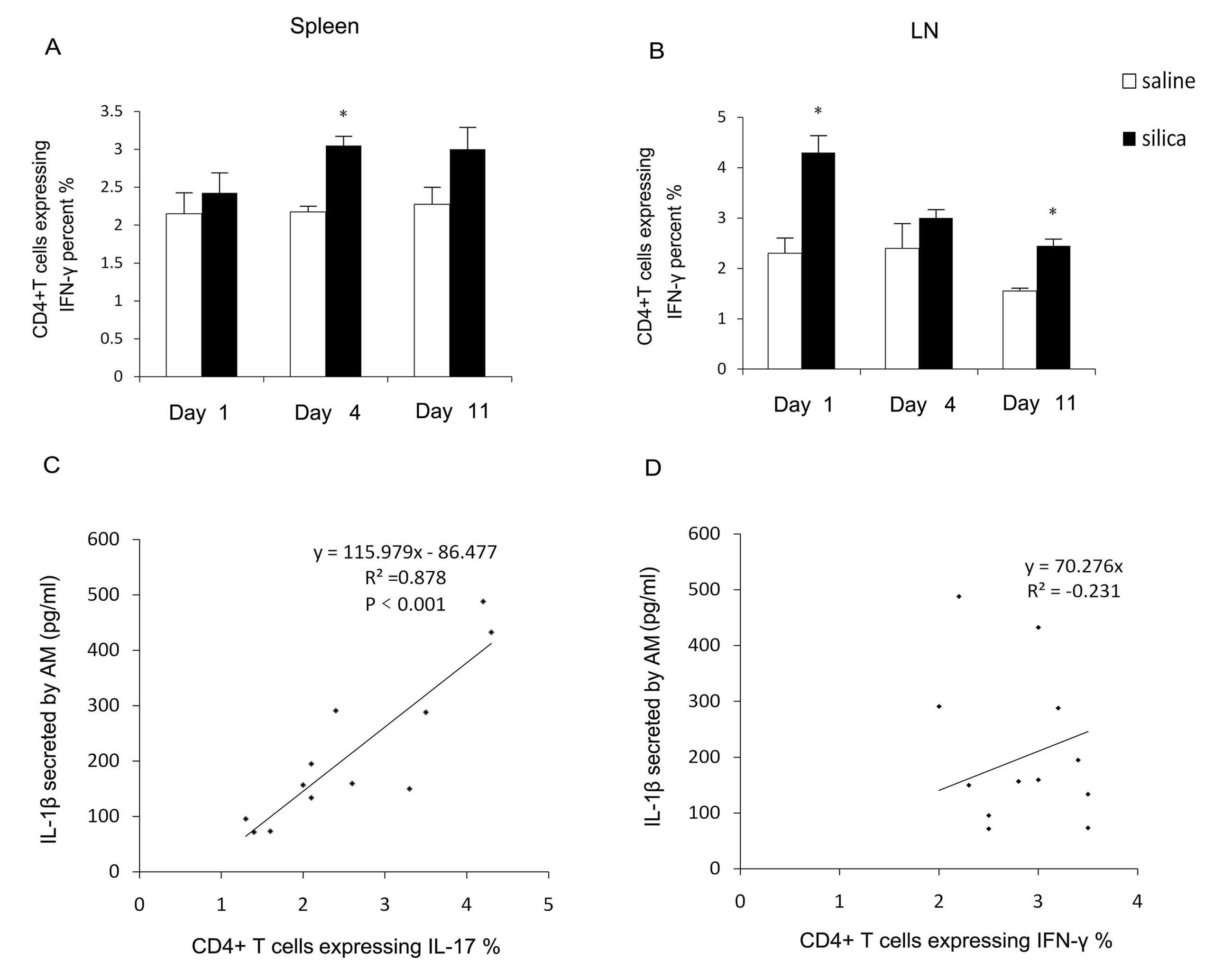

Supplement: Supplementary file 1 — Figure S1 Silica exposure may facilitate a Th1 response in C57/BL6 mice. [file jcmm0018-1773-SD1.tif]

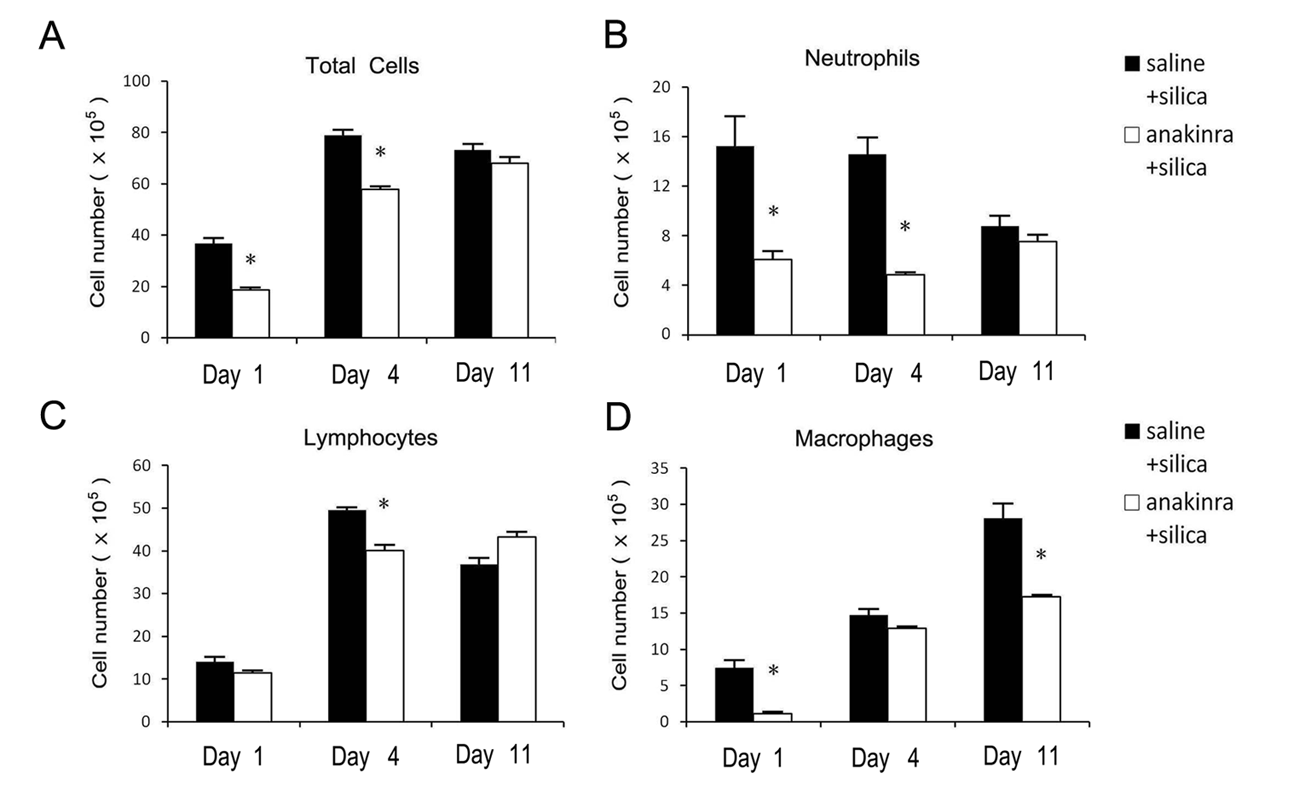

Supplement: Supplementary file 2 — Figure S2 Blockade of the IL-1β receptor by Anakinra decreases the accumulation of BALF inflammatory cells. [file jcmm0018-1773-SD2.tif]

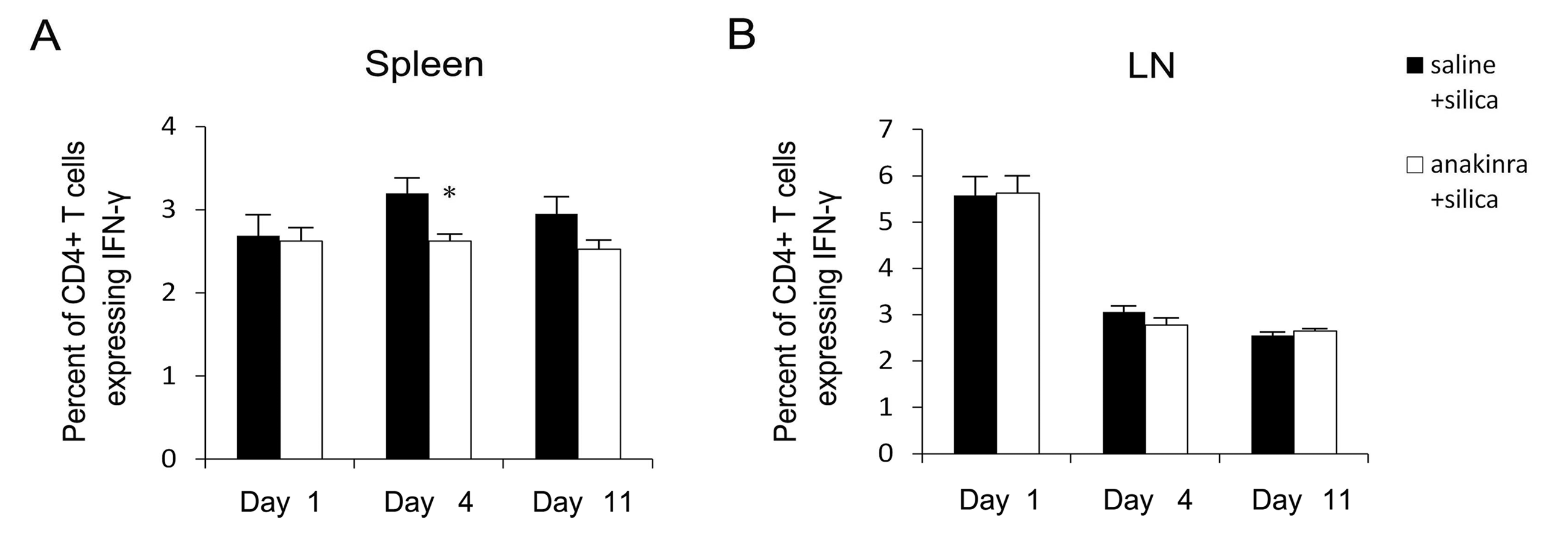

Supplement: Supplementary file 3 — Figure S3 IL-1β does not influence the Th1 immune response in the inflammatory stage of silicosis. [file jcmm0018-1773-SD3.tif]
