# Supplementary material for: Th17 can regulate silica-induced lung inflammation through an IL-1β-dependent mechanism
Source: J Cell Mol Med. 2014 Aug 5;18(9):1773–84. doi: 10.1111/jcmm.12341 (PMC4196653; doi:10.1111/jcmm.12341)
Supplement: Supplementary file 4 — Table S1 Animal grouping moulding and processing. [file jcmm0018-1773-SD4.doc]

Table 1. Animal grouping molding and processing

| **Time** | **silica/saline group** | | **anakinra/saline+silica group** | | | **anti-IL-17mAb/control Ab+silica group** | | |
| --- | --- | --- | --- | --- | --- | --- | --- | --- |
|  | **oral-tracheal** | **sacrifice** | **I.p.** | **oral-tracheal** | **sacrifice** | **i.p.** | **oral-tracheal** | **sacrifice** |
| Day-1 |  |  | anakinra/saline |  |  | 17/control Ab |  |  |
| Day0 | silica/saline |  | anakinra/saline | silica |  |  | silica |  |
| Day1 |  | sacrifice | anakinra/saline |  | sacrifice |  |  | sacrifice |
| Day2 |  |  | anakinra/saline |  |  |  |  |  |
| Day3 |  |  | anakinra/saline |  |  |  |  |  |
| Day4 |  | sacrifice | anakinra/saline |  | sacrifice |  |  | sacrifice |
| Day5 |  |  | anakinra/saline |  |  |  |  |  |
| Day6 |  |  | anakinra/saline |  |  | 17/control Ab |  |  |
| Day7 |  |  | anakinra/saline |  |  |  |  |  |
| Day8 |  |  | anakinra/saline |  |  |  |  |  |
| Day9 |  |  | anakinra/saline |  |  |  |  |  |
| Day10 |  |  | anakinra/saline |  |  |  |  |  |
| Day11 |  | sacrifice | anakinra/saline |  | sacrifice |  |  | Sacrifice |
| Oral-tracheal: oral-tracheal instillation  I.p.: Intraperitoneal injection | | | | | | | | |

All mice were randomly divided into 6 groups as the table above: (group 1/2) exposure to silica by direct oral-tracheal instillation (silica group) or exposure to sterile saline (saline group); (3/4) direct oral-tracheal instillation of silica crystals after the first i.p. administration of anakinra (anakinra + silica group) or after the first i.p. administration of sterile saline (saline + silica group); (5/6) direct oral-tracheal instillation of silica crystals after the first i.p. administration of anti-IL-17 mAb (anti-IL-17 mAb + silica group) or after the first i.p. administration of control Ab (IgG1) (control Ab + silica group). Mice were sacrificed on days 1, 4, and 11 after oral-tracheal instillation.
